# Pathogenicity, Resistance Genes and Integrated Management Strategies of *Potato Virus Y* in Potato

**DOI:** 10.3390/v18030343

**Published:** 2026-03-11

**Authors:** Zijian Zhang, Ran Tian, Kaiqian Wang, Jing Zhou, Haoyu Song, Zizhong Wang, Guixiang Jiao, Yuxiao Du, Haining Huang, Dianqiu Lv

**Affiliations:** 1 College of Agronomy and Biotechnology, Southwest University, Chongqing 400715, China; 2 Integrative Science Center of Germplasm Creation in Western China (CHONGQING) Science City, Southwest University, Chongqing 400715, China; 3 Chongqing Key Laboratory of Potato Biology and Genetic Breeding, Southwest University, Chongqing 400715, China

**Keywords:** Potato virus Y, pathogenic mechanisms, plant immune receptors, *Ry_sto_*, control strategies

## Abstract

*Potato virus Y* (PVY), widely regarded as one of the world’s most important plant viruses, seriously threatens global potato production and food security. PVY deploys its proteins to interact with key host factors, thereby enabling viral replication, accumulation, and systemic infection. PVY also exhibits high genetic diversity and frequent recombination, which promote host adaptation and immune evasion. In response, potato plants perceive viral effectors through intracellular immune receptors and activate antiviral defenses. Over the past decade, significant progress has been made in elucidating PVY–host defense and counter-defense mechanisms. Here, we summarize the molecular basis of PVY pathogenicity and highlight recent advances in PVY resistance genes (e.g., *Ry_sto_* and *Ry_chc_*). Finally, we integrate emerging insights from plant virology and nucleotide-binding leucine-rich repeat (NLR) biology to discuss prospective, multi-pronged strategies for PVY management.

## 1. *Potato Virus Y* and Its Induced Diseases in Potato

*Potato virus Y* (PVY) has a broad host range, covering multiple genera in the *Solanaceae* family, including economically important crops like potato, pepper, tomato and tobacco [1]. The earliest systematic descriptions of PVY date to 1931 [2], and the name *Potato virus Y* was later adopted to distinguish it from *Potato virus X* (PVX) and to reflect the chlorotic symptoms it induces in infected hosts [3]. With advances in virological research and standardized nomenclature, the International Committee on Taxonomy of Viruses (ICTV) classified PVY within the genus *Potyvirus* (family *Potyviridae*) and designated it as the type species of the genus [4].

Based on host responses and interactions with resistance (R) genes, PVY has been classified into several lineages. Among these, three major lineages, including PVY^O^, PVY^C^, and PVY^N^, are widely recognized and have been extensively studied worldwide [5]. Before the 1980s, non-recombinant PVY strains predominated in major potato-producing regions worldwide. Since then, recombinant strains have gained adaptive advantages through the rapid incorporation of parental genomic fragments and have gradually become dominant. Currently, recombinant strains account for more than 90% of PVY isolates in potato-growing regions of Europe, the Americas, Asia, and Africa, whereas non-recombinant strains persist at low frequencies, primarily in parts of South America and Africa characterized by less intensive production systems. Over recent decades, advances in molecular diagnostic techniques have enabled the identification of numerous recombinant PVY variants, most of which are derived from the parental non-recombinant lineages PVY^O^ and PVY^N^. The adaptive benefits conferred by recombination, together with the combined effects of international seed potato trade and aphid-mediated transmission, have driven the emergence and global spread of dominant recombinant strains. Recombination breakpoints are concentrated within three major genomic regions (nt 501–2390, 2391–5850, and 5851–9200) and are frequently reported in North America, Europe, and the Middle East. Host resistance genes (e.g., Ny, Nc, and Nz) recognize specific viral proteins and activate defense responses. However, recombinant strains can modify these recognition targets by incorporating heterologous gene fragments, thereby compromising resistance-mediated immunity. This evolutionary dynamic poses significant challenges for accurate field diagnosis and the implementation of effective integrated disease management strategies [6,7].

Following infection, PVY induces characteristic symptoms including leaf mottling, wrinkling, chlorosis, and systemic necrosis. In potato, PVY can also cause potato tuber necrotic ringspot disease (PTNRD), which markedly reduces tuber marketability and economic value. Symptom severity and type vary across PVY strains and host genotypes. For example, PVY^O^ typically causes mild mosaic or mottling in potato, whereas PVY^C^ can elicit a hypersensitive response (HR) in certain cultivars, manifested as localized or systemic necrosis [3]. Collectively, these disease symptoms impair photosynthesis and assimilate transport, resulting in plant stunting and substantial yield losses [8].

PVY is distributed across all major potato-producing regions worldwide and causes significant economic losses, particularly in North America and Europe. Reported yield losses attributable to PVY can reach up to 80%, and PVY-associated tuber quality defects further reduce market value [9,10].

## 2. Transmission of PVY

PVY is transmitted primarily by aphids in a non-persistent manner, with the green peach aphid (*Myzus persicae*) recognized as the principal vector [11]. During feeding on PVY-infected plants, aphids acquire virions through their piercing–sucking mouthparts. Helper component proteinase (HC-Pro) serves as a key viral factor mediating the non-persistent transmission of potyviruses by aphids. This transmission process relies on specific interactions between HC-Pro, the viral coat protein (CP), and receptors located on aphid mouthparts. The N-terminal KITC motif of HC-Pro binds to receptors in the aphid stylet, enabling transient retention of virions within the stylet. In contrast, the C-terminal PTK motif directly interacts with the highly conserved DAG motif in the CP or its variants, thereby facilitating the association of HC-Pro with viral particles. This coordinated molecular interaction underlies efficient virus acquisition and inoculation by aphids and constitutes the core mechanism by which PVY achieves aphid-mediated transmission [7,12]. Upon transfer to a healthy host, virions retained on the stylet are released during probing and initiate infection [13]. Aphid transmission efficiency is influenced by multiple factors, including aphid species, PVY strain, and environmental conditions such as temperature and humidity. Together, these features of nonpersistent transmission have contributed to the rapid global dissemination of PVY and the substantial losses it causes in potato and other solanaceous crops.

Over the past several decades, PVY population structure has shifted substantially, including the emergence and increasing dominance of recombinant strains, which may be linked to changes in transmission dynamics. Evidence suggests that some PVY strains are transmitted more efficiently by particular aphid species, which may help explain the rapid displacement of earlier strains by newly emerged variants. In parallel, intensified international trade and the expansion of intensive agricultural systems have facilitated the global spread of PVY and its aphid vectors and may also alter aphid population dynamics and feeding behavior, thereby influencing the transmission cycle. These factors likely contribute to the continued prevalence of PVY in many regions despite strict seed certification programs and diverse on-farm management practices.

Also, PVY can persist across seasons and spread over long distances through infected seed tubers, a process often defined as vertical transmission [14]. Infected seed tubers and volunteer plants emerging from tubers left in the field are major sources of primary inoculum that initiate PVY epidemics in successive growing seasons. In asexually propagated potato crops, viral titers can increase with each generation, weakening seed stocks, reducing plant vigor, and ultimately causing substantial yield losses [8]. Accordingly, planting certified seed tubers with low or undetectable PVY levels is an effective strategy to limit PVY spread by reducing primary inoculum and mitigating PVY-associated economic losses [15].

## 3. Molecular Biology and Life Cycle of PVY

### 3.1. PVY Proteins and Their Biological Functions

**Figure 1:**
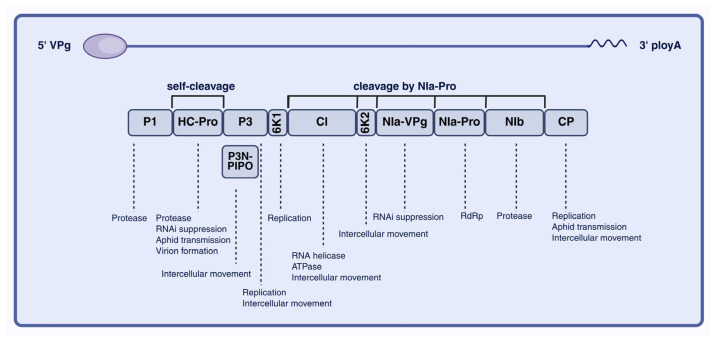
Schematic representation of the PVY genome, polyprotein processing and functions of viral proteins. VPg (viral protein genome-linked), P1 (protein 1), HC-Pro (helper component-proteinase), P3 (protein 3), PIPO (pretty interesting potyviridae ORF), 6K1 (6 kDa protein 1), CI (cytoplasmic inclusion protein), 6K2 (6 kDa protein 2), NIa-VPg (nuclear inclusion a-viral protein genome-linked), NIa-Pro (nuclear inclusion protein A—protease), NIb (nuclear inclusion protein b), CP (coat protein), poly A (poly A tail). RNA-dependent RNA polymerase (RdRp). Image created using BioRender.com, with permission.

PVY virions are flexuous, non-enveloped filaments approximately 700 nm in length and 11–13 nm in diameter, with a helical pitch of 3.4–3.5 nm. The virion surface is composed of more than 2000 copies of approximately 30 kDa CP. The PVY genome is a positive-sense single-stranded RNA of approximately 9.7 kb, bearing a covalently linked viral protein genome-linked (VPg) at the 5′ end and a poly(A) tail at the 3′ end (Figure 1). Cryo-electron microscopy resolved the PVY virion structure and showed that the capsid adopts left-handed helical symmetry, with approximately 8.8 CP subunits per helical turn. Notably, the N- and C-terminal regions of CP display conformational plasticity, which likely underpins the functional versatility of PVY during infection [16].

PVY gene expression follows the canonical potyviral strategy of polyprotein processing. The genome contains a single long open reading frame (ORF) that is translated into a polyprotein precursor, which is subsequently cleaved by virus-encoded proteinases to generate mature functional proteins. The NIa protease mediates most cleavage events, producing both nonstructural and structural proteins, including P1, HC-Pro, P3, CI, NIa, NIb, and CP (Figure 1) [17].

**Figure 2:**
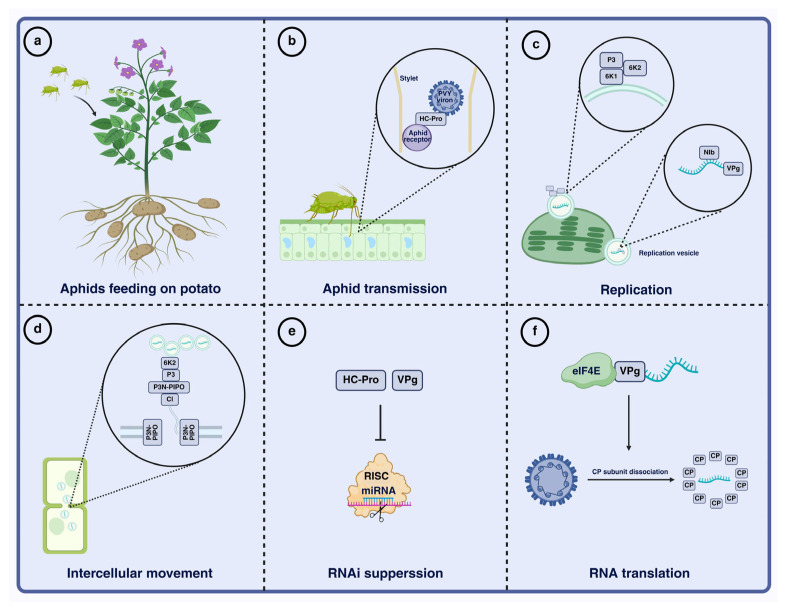
An overview of PVY infection processes. (**a**) PVY-carrying aphids feed on potato. (**b**) Aphid transmission of PVY. The aphid stylet tip is enlarged to show the interaction between the N-terminal of HC-Pro binding to the aphid receptor and the C-terminal binding to CP. (**c**) Viral replication. Upon entry into the host, PVY undergoes translation. The viral RNA is amplified within replication vesicles by NIb. NIb modifies VPg, enabling its covalent binding to vRNA. The replication vesicles, consisting of 6K2, 6K1, and P3, are tightly associated and ultimately accumulate near chloroplasts. (**d**) Cell-to-cell movement of PVY. The interaction of 6K2, P3, P3N-PIPO, and CI facilitates the recruitment of replication vesicles to plasmodesmata. P3N-PIPO anchors the CI conical inclusions to plasmodesmata, enabling viral movement between cells. (**e**) RNAi suppression. HC-Pro and VPg suppress host RNA silencing mechanisms, protecting replicating vRNA from antiviral responses. (**f**) Viral translation. VPg binds directly to eIF4E to initiate translation. The interaction between VPg and eIF4E enables preferential translation of viral RNA over host mRNA. The force generated during translation leads to the removal of CP subunits. Image created using BioRender.com, with permission.

As shown in Figure 1, P1 is the first protein encoded by the 5′ region of the PVY genome and functions as a serine protease with a relatively high positive charge. P1 exhibits autocatalytic protease activity and cooperates with HC-Pro and NIa-Pro to mediate cleavage and processing of the viral polyprotein [18]. In addition, P1 forms a complex with HC-Pro and VPg that interacts with the host translation initiation factors eIF4E and eIF(iso)4E, thereby promoting preferential translation of viral RNA while suppressing host mRNA translation [19]. Furthermore, P1 associates with HC-Pro and P3N-PIPO to generate the short P1–HC-Pro–P3N-PIPO polyprotein, which provides a structural basis for viral cell-to-cell movement. The next protein, HC-Pro, is a cysteine protease that undergoes autocleavage at its C terminus and facilitates CP binding to aphid receptors by forming a reversible molecular bridge to influence aphid transmission efficiency (Figure 2a,b). HC-Pro binds to the host translation initiation factors eIF4E and eIF(iso)4E, promoting preferential translation of viral RNA (vRNA) while suppressing host mRNA translation [20]. It also participates in the formation of virus-induced particles (PGs) and directly interacts with the CP, contributing to the stable assembly of virions. In addition, interaction with host VARICOSE (VCS) proteins regulates virion maturation and long-distance movement [21,22]. HC-Pro further cooperates with other viral proteins to modulate viral replication and cell-to-cell movement and suppresses the host antiviral RNA silencing pathway through multiple mechanisms [23]. P3 contains hydrophobic domains at both the N and C termini and may contribute to intercellular movement in concert with P3N-PIPO [24]. The small protein 6K1 is required during the early stages of viral replication and can reduce plant protease activity and modulate transcripts associated with jasmonic acid biosynthesis, thereby promoting infection [25,26]. Additional PVY proteins coordinate replication and movement. CI is a multifunctional helicase that contributes to viral replication and may also interact with P3N-PIPO to facilitate intercellular movement and long-distance transport [24]. 6K2 induces ER-derived replication vesicles that traffic to chloroplasts, supporting efficient viral replication [26]. NIa comprises the genome-linked protein (VPg) and the NIa protease (NIa-Pro), with NIa-Pro mediating most polyprotein cleavage events. NIb is the RNA-dependent RNA polymerase (RdRp) and recruits host proteins to the viral replication complex (VRC), thereby promoting genome replication [7]. In addition, NIb can be SUMOylated by SUMO3, which shifts its localization from the nucleus to the cytoplasm and promotes infection [27]. Finally, CP is the major structural component of the virion and is indispensable for cell-to-cell movement, although its activity depends on cis expression [28].

### 3.2. PVY Infection Cycle

After successful evasion of host defenses, PVY establishes systemic infection through coordinated replication and movement. Replication occurs on host endomembrane structures, with ER-derived membranes playing a prominent role. During infection, 6K2 induces ER membrane curvature and promotes the formation of replication vesicles that provide a specialized microenvironment for viral RNA synthesis. Multiple viral proteins, including NIb, CI, P3, and 6K2, are recruited to these sites and assemble with VPg and NIa-Pro to form the replication complex (Figure 2c). This virus-induced organelle remodeling not only enhances replication efficiency but may also reduce immune detection [29,30].

Systemic infection further depends on efficient cell-to-cell and long-distance movement. CI forms tubular structures at plasmodesmata, facilitating intercellular transport of viral RNA and/or virions. P3N-PIPO is also critical for cell-to-cell movement because it localizes to plasmodesmata and modulates their permeability (Figure 2d) [31]. In addition, CP contributes to long-distance transport through the phloem. Together, movement efficiency influences the rate and severity of symptom development and represents a key determinant of PVY pathogenicity.

PVY pathogenicity is further shaped by high genetic variability and frequent recombination. Amino acid variation in proteins such as HC-Pro, P3, and CP can markedly influence RNA-silencing suppression, necrosis induction, and the establishment of systemic infection. The emergence of the recombinant strain PVY^NTN^ is widely regarded as a notable outcome of PVY evolution and adaptation to new host environments [32,33].

## 4. Molecular Mechanisms of PVY Counter-Defense Against Host Immunity

RNA silencing is a central component of antiviral defense in plants. Viral RNAs are recognized and processed into small interfering RNAs (siRNAs) by Dicer-like proteins; these siRNAs then guide the RNA-induced silencing complex (RISC) to target and degrade viral RNA [12,34]. The PVY suppressor HC-Pro can bind siRNAs and miRNAs, thereby preventing their loading onto ARGONAUTE (AGO) proteins and inhibiting RISC assembly (Figure 2e) [35]. HC-Pro also perturbs host miRNA homeostasis, disrupting developmental regulation and contributing to symptoms such as mosaicism and leaf malformation during PVY infection [36]. Consistent with these roles, HC-Pro loss-of-function or mutation markedly attenuates PVY pathogenicity and can compromise systemic infection [37]. Beyond HC-Pro, the PVY 6K1 protein can competitively bind 14-3-3 proteins and disrupt their interaction with the defense-related factor translationally controlled tumor protein (TCTP), thereby weakening antiviral immunity [38].

In addition to suppressing RNA silencing, PVY can exacerbate disease symptoms by triggering an exaggerated host immune response. Certain PVY strains (e.g., PVY^N^ and PVY^NTN^) induce localized or systemic necrosis in susceptible host genotypes [39]. This outcome is often associated with recognition of viral effectors by nucleotide-binding leucine-rich repeat (NLR) proteins. In particular, motifs within HC-Pro, CP, or P3 may be perceived by host NLR, activating effector-triggered immunity (ETI) and programmed cell death (PCD) [40]. When immune activation is excessive or poorly contained, it can contribute directly to severe pathology, including systemic necrosis or tuber necrosis [41].

PVY pathogenicity also depends on interactions with key host factors. The translation initiation factors eIF4E and eIF(iso)4E are essential for potyvirus infection, and VPg interacts with these factors to promote translation of viral RNA (Figure 2f) [42]. Accordingly, mutations in these host factors can confer recessive resistance to PVY, underscoring the central role of PVY–host factor interactions in disease development [43].

## 5. Potato NLRs Mediating Resistance to PVY

### 5.1. Structure and Types of Plant NLRs

Plants have evolved a sophisticated innate immune system to detect and restrict microbial invasion [44,45,46]. This immune system is commonly described as comprising two interconnected layers: pattern-triggered immunity (PTI) and ETI. PTI constitutes the first line of defense and is initiated when cell-surface pattern recognition receptors (PRRs) perceive pathogen- or microbe-associated molecular patterns (PAMPs/MAMPs) or damage-associated molecular patterns (DAMPs). During coevolution with their hosts, pathogens deploy effector proteins to suppress PTI and promote infection. In turn, plants encode intracellular NLRs that recognize these effectors, thereby activating the typically stronger ETI response [45,46].

NLRs typically comprise a central nucleotide-binding (NB-ARC) domain, a C-terminal leucine-rich repeat (LRR) domain, and an N-terminal signaling domain, usually a Toll/interleukin-1 receptor (TIR) or coiled-coil (CC) domain [47]. Based on the N-terminal domain, plant NLRs are commonly classified into three groups: TIR-NLRs (TNLs), CC-NLRs (CNLs), and RPW8-NLRs (RNLs) [48]. In addition, some NLRs carry integrated noncanonical domains that broaden recognition specificity and diversify functional output [49,50,51]. NLRs detect pathogen effectors either directly or indirectly; indirect recognition includes guard, bait, and integrated-bait models in which NLRs sense effector-induced perturbations of host targets or decoy proteins [52].

### 5.2. PVY Resistance in Potato: Ry Genes and Ny Genes

Over recent decades, researchers have screened wild potato germplasm for PVY resistance and identified associated resistance genes. Two major resistance phenotypes have been described in potato: extreme resistance (ER) and HR. ER, typically conferred by Ry genes, is characterized by broad-spectrum, durable resistance and often an absence of visible symptoms after inoculation [53]. In contrast, HR, commonly mediated by Ny genes, involves localized programmed cell death but may fail to restrict systemic viral movement under certain conditions, allowing PVY infection to proceed [54].

PVY resistance genes have been introgressed into cultivated potato from wild and domesticated *Solanum* species. To date, ten distinct resistance loci have been mapped to specific potato chromosomes, although only a subset has been cloned and characterized in detail (Table 1) [55]. Five loci confer ER: *Ry_adg_* on chromosome XI [56]; *Ry_sto_* and *Ry-f_sto_* on the short arm of chromosome XII [41,53,56,57,58,59,60]; and *Ry_chc_* on chromosome IX [58,59]. The remaining five loci confer HR: *Ny-1* [60] and *Ny-Smira* [61] on chromosome IX; *Ny-2* [62] on chromosome XI; and *Ny_tbr_* and *Ny_spl_* on chromosome IV [63,64]. Among the Ry genes, the two cloned members are *Ry_sto_* and *Ry_chc_*, both encoding TIR-NLR (TNL) immune receptors.

The *Ry_sto_* gene, originally derived from the wild species *Solanum stoloniferum*, has been introgressed into several potato cultivars (e.g., ‘Alicja’, ‘Bzura’, and ‘White Lady’) through classical breeding and confers ER to diverse PVY strains [66]. Using modern genomics approaches, including resistance gene enrichment sequencing (RenSeq) and PacBio single-molecule real-time (SMRT) sequencing, *Ry_sto_* was isolated and cloned. Functional analyses indicate that Ry_sto_ encodes a typical TNL receptor and that Ry_sto_-mediated ER requires signaling components such as EDS1 and NRG1. Previous work has shown that helper NLR ADR1 participates in TIR-NLR immune signaling, so its functional involvement in Ry_sto_-mediated antiviral pathways requires further validation [67]. The conserved co-chaperone complex (SGT1, RAR1, HSP90), which maintains NLR folding and stability in plant immunity, may also function in Rysto-mediated resistance [68]. In addition, Ry_sto_ expression is likely negatively regulated by microRNAs (miRNAs) via mRNA degradation, translational inhibition, or epigenetic transcriptional repression [69]. Unlike the HR conferred by *Ny*-1, which breaks down at 28 °C and permits systemic infection, *Ry*_sto_-mediated resistance remains effective at elevated temperatures. This temperature stability has been attributed to stable intermolecular interactions between the WHD subdomain of the NB-ARC domain and surface residues of the adjacent LRR domain, which preserve ligand binding and oligomerization required for defense activation [70]. Mechanistically, Ry_sto_ directly recognizes the PVY CP, and this recognition depends on correct folding of the CP core region (CP_77-226_), particularly the conserved RNA-binding pocket. Point mutations that disrupt this core architecture markedly reduce or abolish Ry_sto_ binding and resistance. Consistent with this mechanism, Ry_sto_ can also recognize CPs from other *Potyviridae* members (e.g., *plum pox virus* and *turnip mosaic virus*) and confer resistance in *Solanaceae* and *Brassicaceae*, highlighting its potential for cross-species deployment [71]. Large-scale surveys of wild potato relatives have identified abundant Ry_sto_-like allelic variation: the TIR and NB-ARC domains are comparatively conserved, whereas the LRR domain is more diverse, providing valuable resources for future resistance breeding [72].

The *Ry_chc_* gene originates from the wild diploid potato species *Solanum chacoense*. It was cloned through fine mapping and functional complementation. Like Ry_sto_, Ry_chc_ encodes a TIR-NLR (TNL) immune receptor and confers resistance to multiple PVY strains in both diploid and tetraploid potato backgrounds [73,74]. Evolutionary analyses of the *Ry_chc_* locus revealed frequent sequence exchange and signatures of positive selection, consistent with rapid diversification during coevolution with PVY. Molecular markers developed from the *Ry_chc_* sequence reportedly achieve high accuracy in predicting resistance phenotypes, providing a useful tool for marker-assisted selection [74]. Notably, recent diversity surveys found that although Ry_chc_ shows relatively low sequence variation among *S. chacoense* accessions, specific single-nucleotide polymorphisms (SNPs) generate loss-of-function alleles; this likely explains why some carriers remain fully susceptible to PVY [75]. These findings highlight the importance of developing markers that distinguish functional alleles rather than simply detecting gene presence.

## 6. Future Multifaceted PVY Control Strategies: From Molecular Breeding to Field Application

**Figure 3:**
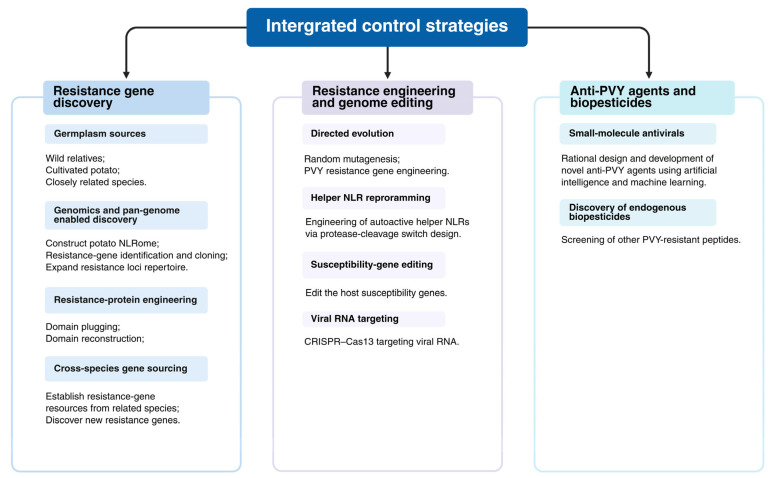
Multiple Control Strategies for PVY. Image created using BioRender.com, with permission.

Developing comprehensive PVY resistance gene resources spanning wild relatives, cultivated potato, and closely related species is central to achieving durable genetic resistance (Figure 3). With rapid advances in genomics and pan-genomics, researchers can systematically identify and clone novel resistance genes by constructing a potato “NLRome” across wild and cultivated germplasm, thereby expanding the pool of resistance loci. In parallel, protein-engineering strategies such as domain plugging and domain reconstruction provide feasible routes for targeted optimization of resistance proteins, potentially broadening recognition specificity and improving resistance durability [76]. Beyond potato itself, establishing resistance gene resources from closely related species may further enable the discovery of effective, durable, and broad-spectrum resistance genes [77].

In-depth research on plant NLRs has also provided a foundation for rational engineering of disease resistance genes (Figure 3). Directed evolution approaches, such as random mutagenesis of resistance genes including Rx, Sw-5b, and Tm-2^2^, have shown that substitutions at specific amino acid positions can broaden the resistance spectrum [78,79,80]. Using the directed evolution strategy, random mutagenesis of PVY resistance genes (e.g., Ny-1) can generate variants with stable PVY resistance under high-temperature stress. Coupled with advances in genome editing, targeted point mutations can be introduced using base-editing technologies to generate improved resistance alleles and germplasm. Beyond modifying sensor NLRs, reprogramming helper NLR activation has emerged as a promising strategy. For example, Wang et al. fused pathogen protease cleavage sites to autoactive helper NLRs to create engineered autoactive NLRs (aNLRs) that remain inactive under normal conditions but are activated upon protease-mediated cleavage during infection, thereby triggering a strong and potentially broader immune response [81]. In principle, this approach could be combined with genome editing to modify helper NLR and enhance resistance to diverse pathogens, including viruses, fungi, bacteria, and nematodes. In addition, editing host susceptibility genes such as eIF4E and GSL5 has been reported to increase resistance to PVY and clubroot, respectively [82,83]. Because susceptibility genes are endogenous to plants, this strategy can be comparatively straightforward to implement and broadly applicable. Finally, CRISPR–Cas13-based RNA targeting has been used to generate PVY-resistant germplasm, providing an additional route for resistance improvement [84]. Compared with RNAi, CRISPR-Cas13 exhibits more rigorous target recognition specificity, significantly reduced off-target effects, and higher efficiency, as it does not rely on the host’s endogenous RNA silencing machinery.

To date, the mainstream commercially available antiviral agents for field-based control of PVY are primarily amino-oligosaccharins, ribavirin, and ningnanmycin. However, these commercial agents have notable limitations of varying severity, including suboptimal field control efficacy and unfavourable economic cost-effectiveness for large-scale application. As research progresses, anti-PVY discovery is shifting from empirical screening toward structure- and mechanism-guided design (Figure 3). Rational design of targeted antiviral compounds against core processes of the PVY infection cycle, including viral genome replication, virion assembly, intercellular movement, and systemic long-distance movement, is critical to enhancing the target specificity and field control efficacy of chemical-based PVY management. Wei et al. (2024) [85] synthesized a novel aryl imidazodiazepine scaffold via a carbene-catalyzed tri- and tetracycloaddition reaction and reported strong activity against PVY. Further analyses indicated that compound 3j competitively inhibits the interaction between PVY CP and NtCPIP, thereby attenuating systemic infection [85]. In addition, 1,3,5-triazine derivatives bearing piperazine moieties have shown anti-PVY activity, expanding the chemical diversity of candidate antiviral agents [86]. Recent studies have also shown that endogenous plant peptides (e.g., APP3-14, GmPEP914, GmPEP890, and PSKα) can enhance disease control in several crop–pathogen systems, including citrus Huanglongbing, soybean diseases, and rice blast [87,88,89]. In addition, the PAMP-induced potato peptide StPIP1 enhances host resistance against PVY [90]. Artificial intelligence (AI)-assisted rational design is a key future direction for anti-PVY agent development. Integrating cutting-edge PVY control research with deep learning models enables efficient discovery of novel active molecules, with improved prediction of anti-PVY activity and compound druggability. Development of plant immune activators to boost host intrinsic defenses and creation of supramolecular assembly technology to optimize the physicochemical properties and field persistence of anti-PVY agents are also highly promising avenues. Future PVY control research will prioritize multidisciplinary collaboration, shifting from empirical single-compound screening to systems biology-based rational drug design, to deliver more efficient, safe, and eco-friendly novel anti-PVY agents.

## 7. Conclusions

PVY is among the most destructive plant viruses in major potato-producing regions worldwide and causes substantial economic losses to potato production. PVY regulates key stages of its life cycle through interactions between its encoded proteins and host factors. These interactions also reprogram the host intracellular environment to facilitate infection and ultimately enable systemic spread. Despite significant advances, many mechanistic questions remain unresolved. In particular, the spatiotemporal molecular contexts in which PVY-encoded proteins interact with host factors, as well as the functional impacts of these interactions on viral infection efficiency and host physiological homeostasis, remain to be fully elucidated. Moreover, the coordination and synergistic regulation of fundamental biological processes, including viral replication, translation, and suppression of host immunity, represent important areas for investigation. Several core questions arise from these gaps in knowledge. Does the maturation of replication vesicles and their fusion with chloroplasts promote viral protein translation and suppression of antiviral pathways? Do distinct stages of replication vesicle maturation transmit retrograde signals to the nucleus, thereby remodeling the cellular environment to favor infection? How is the PVY genome released from replication vesicles and stabilized for subsequent cell-to-cell movement? Emerging evidence indicates that certain host proteins interacting with viral components bind PVY RNA and may have co-evolved to facilitate both viral protein synthesis and virion assembly. However, the mechanistic links among PVY replication, translation, and particle formation, as well as the molecular basis and spatiotemporal regulation of capsid assembly, remain poorly understood. Elucidating the interaction network between PVY-encoded proteins and host factors, and defining the molecular mechanisms governing viral infection and host immunity, will provide precise targets for resistance breeding in potato. Such insights will also establish a theoretical framework for the development of novel broad-spectrum antiviral strategies, underscoring both their scientific significance and practical value.

## Figures and Tables

**Table 1:** PVY resistance genes and their resistance gene response, chromosome location, spectrum of resistance, origin, ploidy level and reference.

Resistance Gene	Response	Chromosome Location	Spectrum of Resistance	Origin	Ploidy Level	References
Nc_spl_	HR	IV	PVY^C^	*Solanum sparsipilum*	2×	Moury et al. (2011) [64]
Nc_tbr_	HR	Not reported	PVY^C^	*S. tuberosum*	4×	Moury et al. (2011) [64]
Ny_tbr_	HR	IV	PVY^O^	*S. tuberosum*	4×	Moury et al. (2011) [64]
Ny_-Smira_	HR	IX	PVY^NTN^	*S. tuberosum*	4×	Tomczyńska et al. (2014) [61]
Ny-1	HR	IX	PVY^O^ and PVY^N^	*S. demissum*	6×	Szajko et al. (2007) [60]
Ny-2	HR	XI	PVY^N-Wi^	*S. chacoense*	2×	Szajko et al. (2014) [62]
Ry_chc_	ER	IX	All	*S. chacoense*	2×	Hosaka et al. (2001)[58]; Sato et al. (2006) [59]
Ry_adg_	ER	XI	All	*S. tuberosum* ssp. *adg*	2×	Hämäläinen et al. (1997) [56]
Ry_sto_	ER	XII	All	*S. stoloniferum*	4×	Song et al. (2006) [65]; Cernák et al. (2008) [57]
Ry-*f*_sto_	ER	XII	All	*S. stoloniferum*	4×	Flis et al. (2005) [53]

HR, hypersensitive response; ER, extreme resistance.

## Data Availability

No new data were created or analyzed in this study.
